# Association of Red Cell Distribution Width-Coefficient of Variation (RDW-CV) and RDW-Standard Deviation (RDW-SD) With Clinical Severity and Short-Term Outcomes in Patients With Acute Coronary Syndrome: A Prospective Observational Study

**DOI:** 10.7759/cureus.114035

**Published:** 2026-08-05

**Authors:** Devendra Ahirwar, Hindeshwari Rai, Neha Prasad, Shikha Agarwal

**Affiliations:** 1 Internal Medicine, Bundelkhand Medical College, Sagar, IND; 2 Medicine, Bundelkhand Medical College, Sagar, IND; 3 Nephrology, All India Institute of Medical Sciences, Bibinagar, IND; 4 Pathology and Laboratory Medicine, Bundelkhand Medical College, Sagar, IND

**Keywords:** acute coronary syndrome, grace score, killip class, mortality, red cell distribution width, red cell distribution width-coefficient of variation (rdw-cv)

## Abstract

Background: Acute coronary syndrome (ACS) is a major cause of cardiovascular morbidity and mortality. Red cell distribution width-coefficient of variation (RDW-CV), a routinely available complete blood count parameter, has emerged as a potential prognostic marker in cardiovascular diseases.

Aim and objective: This study aimed to assess the prognostic significance of RDW in patients with ACS.

Materials and methods: This prospective observational study was conducted in the Department of Medicine at Bundelkhand Medical College and Hospital, Sagar, Madhya Pradesh, India. A total of 210 patients diagnosed with ACS were included. Demographic details, risk factors, clinical history, RDW-CV, red cell distribution width-standard deviation (RDW-SD), ECG findings, troponin I status, Killip class, Global Registry of Acute Coronary Events (GRACE) score, hospital stay, and mortality were analyzed. Statistical analysis was performed using IBM SPSS Statistics software, version 25 (IBM Corp., Armonk, NY, USA), and p<0.05 was considered statistically significant.

Results: Among 210 patients, 141 (67.10%) were male, and 69 (32.85%) were female. The mean age was 57.49 ± 12.45 years, and most patients belonged to the 40-60-year age group, 105 (50.00%). Smoking was reported in 48 patients (22.85%), followed by hypertension in 47 (22.40%) and diabetes mellitus in 45 (21.42%). On Day 1, RDW-CV was elevated in 111 patients (52.86%). In-hospital mortality occurred in 10 patients (4.8%); nine out of 111 patients (8.1%) in the high RDW-CV group and one out of 99 patients (1.0%) in the low RDW-CV group died, showing a significant association between elevated RDW-CV and mortality (p<0.05). Mean RDW-CV increased progressively with higher Killip class, from 13.8 ± 2.1 in Class I to 16.6 ± 1.6 in Class IV (p < 0.001). GRACE scores also increased significantly with higher Killip class (p < 0.0001).

Conclusion: RDW-CV was significantly associated with mortality, Killip class, and GRACE score in patients with ACS. It may serve as a simple, inexpensive, and routinely available prognostic marker for assessing disease severity and clinical outcome in ACS patients.

## Introduction

Acute coronary syndrome (ACS) encompasses unstable angina, non-ST-segment elevation myocardial infarction, and ST-segment elevation myocardial infarction. It typically results from acute plaque rupture or erosion with superimposed thrombosis, leading to partial or complete obstruction of coronary blood flow [1,2]. Despite significant advances in diagnostic modalities, pharmacological therapies, and revascularization strategies, ACS remains a major cause of morbidity and mortality worldwide, particularly in resource-limited settings where delayed presentation and limited access to advanced cardiac care may adversely affect outcomes [3,4].

Early risk stratification is crucial in the management of ACS, as patients present with varying degrees of clinical severity, hemodynamic compromise, myocardial injury, and risk of complications. Established clinical tools such as the Killip classification and the Global Registry of Acute Coronary Events (GRACE) score are widely used for prognostication; however, they require integrating multiple clinical, biochemical, and electrocardiographic parameters [5,6]. Consequently, there is growing interest in identifying simple, cost-effective, and readily available biomarkers to aid early risk assessment and guide clinical decision-making [7].

Red cell distribution width (RDW), a routinely reported parameter in the complete blood count, reflects the variability in erythrocyte size. Although traditionally used to evaluate anemia, elevated RDW has increasingly been recognized as a marker of systemic inflammation, oxidative stress, impaired erythropoiesis, and endothelial dysfunction [8,9]. These pathophysiological processes play a significant role in the development and progression of atherosclerosis, plaque instability, myocardial ischemia, and heart failure in patients with ACS. Several studies have demonstrated an association between elevated RDW and adverse cardiovascular outcomes, including increased mortality and major adverse cardiac events [10].

In this context, the present study was undertaken to evaluate the prognostic significance of RDW in patients with ACS and to examine its association with mortality, Killip class, GRACE score, and other clinical outcomes.

## Materials and methods

The present prospective observational study was conducted as part of a postgraduate thesis in the Department of Medicine, Bundelkhand Medical College and Hospital, Sagar, Madhya Pradesh, India. The study was conducted over 18 months, from 10 March 2023 to 9 September 2024, after approval from the Institutional Ethics Committee, Bundelkhand Medical College, Sagar, Madhya Pradesh, India, vide approval certificate number IECBMC/2023/124 dated 10 March 2023. Written informed consent was obtained from all participants before enrolment.

**Figure 1:**
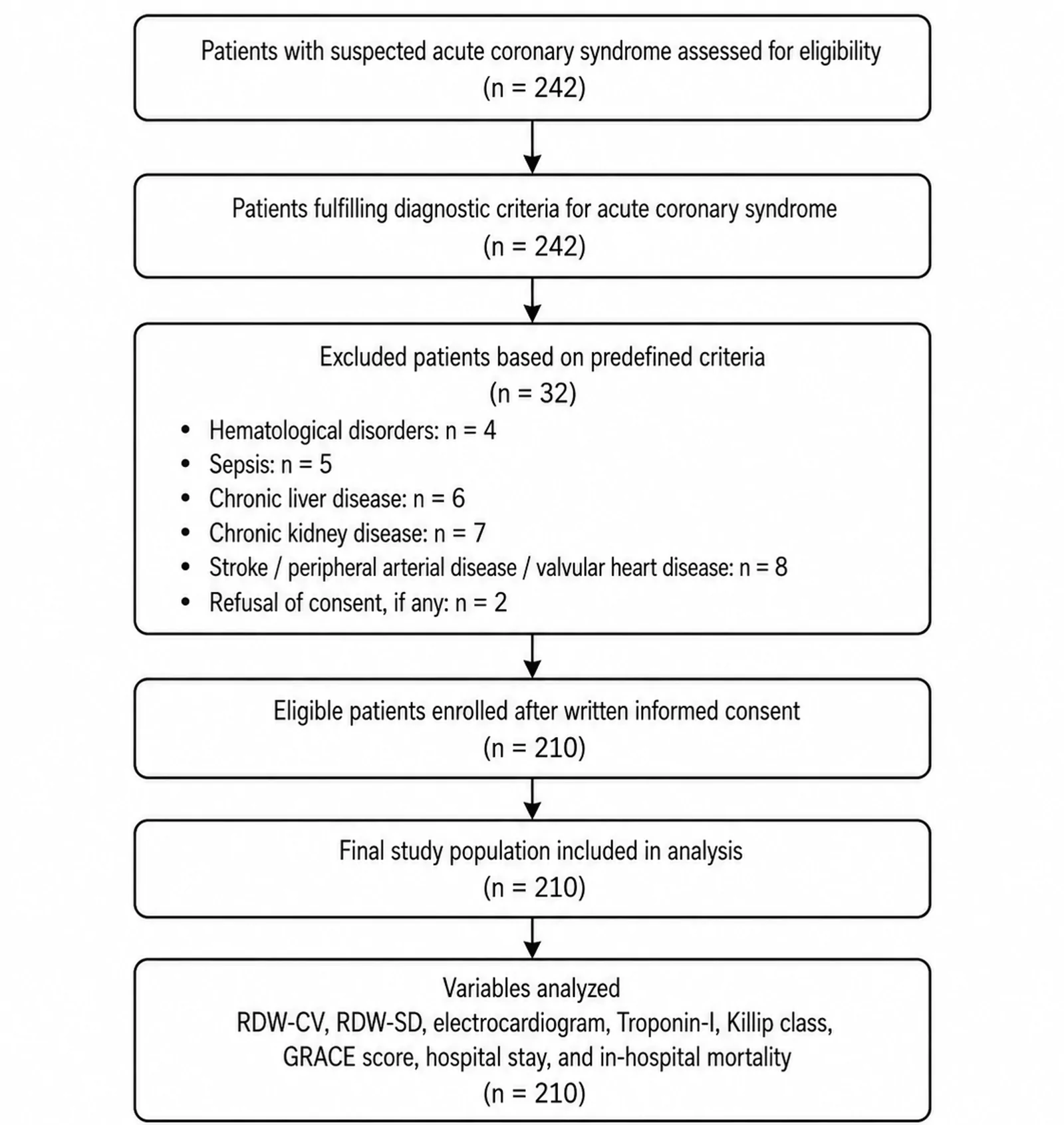
Flow diagram showing the screening, exclusion, enrolment, and final analysis of patients with acute coronary syndrome RDW-CV, red cell distribution width-coefficient of variation; RDW-SD, red cell distribution width-standard deviation; GRACE, Global Registry of Acute Coronary Events

The study population consisted of patients diagnosed with ACS. Patients were recruited after confirming eligibility according to predefined inclusion and exclusion criteria. Patients with ACS who provided informed consent were included in the study. Patients with hematological disorders, sepsis, chronic liver disease, valvular heart disease, chronic kidney disease, stroke, and peripheral arterial disease were excluded, as these conditions could independently affect red cell distribution width or clinical outcome and thereby confound the study findings (Figure 1). Patients with a previously diagnosed hematological disorder were excluded. Hemoglobin and routine complete blood count parameters were recorded; however, systematic biochemical screening for iron, vitamin B12, and folate deficiencies was not performed. Therefore, subclinical or previously undiagnosed nutritional deficiencies could not be completely excluded.

A total of 210 patients with ACS were included in the final analysis.

Sample size was calculated using the following formula:

$n = \frac{Z^{2} \times p \times q}{d^{2}}$

where Z = 1.96 (for 95% confidence level), p = 14%, q = 86% (100 − p), d = 5% (allowable error)

The calculated sample size was 185. After adding 10% for possible loss to follow-up, the required sample size became 204. In this study, a total of 210 eligible patients were included in the final analysis.

Data were collected using a specially designed restructured proforma and case record format (Appendix A). Detailed clinical history, demographic details, presenting complaints, past medical history, risk factors, medication history, and relevant clinical findings were recorded for each patient. Blood investigations, including complete blood count parameters (RDW-CV and RDW-SD), cardiac biomarkers, and electrocardiographic findings, were documented. Patients were assessed clinically during hospitalization, and the in-hospital course was evaluated for length of hospital stay, mortality, and cardiovascular complications.

The main study variable was RDW, assessed as RDW-CV and RDW-SD. RDW values were evaluated in relation to the severity and outcomes of ACS. RDW-CV was categorized as normal/low (≤14.5%) and elevated (>14.5%) according to the reference range used by the institutional laboratory. Complete blood count parameters, including RDW-CV and RDW-SD, were measured using the Z3 automated hematology analyzer (Zybio Inc., Chongqing, China). The same analyzer and institutional laboratory reference ranges were used throughout the study period. Clinical severity was assessed using the Killip classification and the GRACE score. The Killip classification was used as a bedside clinical grading system for heart failure severity in acute myocardial infarction, as originally described by Killip and Kimball [11]. The GRACE score was used for ACS risk stratification based on the GRACE model described by Granger et al. [5]. ECG findings such as T-wave inversion and Q waves, troponin-I status, duration of hospital stay, and mortality were also analyzed. RDW-CV was measured at Day 1, Day 5, and one-month follow-up to assess its trend over time and its relationship with clinical severity.

Statistical analysis

The collected data were entered into Microsoft Excel 2010 (Microsoft Corp., Redmond, WA, USA), and statistical analysis was performed using IBM SPSS Statistics software, version 25 (IBM Corp., Armonk, NY, USA). Continuous variables were expressed as the mean with standard deviation or the median, depending on the data distribution. Categorical variables were expressed as frequency and percentage. Group comparisons were performed using the independent t-test for parametric data and the Mann-Whitney U test for nonparametric data. Paired comparisons were performed using a paired t-test for parametric data and a Wilcoxon signed-rank test for nonparametric data. For comparison among more than two groups, one-way analysis of variance or the Kruskal-Wallis test was applied, as appropriate. Chi-square test was used for categorical variables. Spearman’s rho was used to assess the association between RDW status and GRACE score. Receiver operating characteristic analysis was performed to evaluate the diagnostic performance of RDW for the Killip class. A p-value of less than 0.05 was considered statistically significant.

## Results

A total of 210 patients diagnosed with ACS were included in the final analysis. The demographic and baseline clinical profile of the study population is summarized in Table 1. The mean age was 57.49 ± 12.45 years, and most patients were in the 40-60 years age group (105/210, 50.0%), followed by those aged >60 years (80/210, 38.1%). Males constituted 141 patients (67.1%), showing male predominance. Hypertension, diabetes mellitus, and smoking were the most frequently associated risk factors. Troponin-I was positive in 152 patients (72.4%). T-wave inversion was the most frequent ECG finding, observed in 137 patients (65.2%), followed by ST-segment elevation in 129 (61.4%).

**Table 1:** Characteristics of the study population Data are presented as frequency (percentage) unless otherwise specified. Continuous variables are expressed as mean ± standard deviation. Percentages were calculated using the total study population (N = 210) as the denominator. SD, standard deviation; COPD, chronic obstructive pulmonary disease; ACE, angiotensin-converting enzyme; ARB, angiotensin receptor blocker; ECG, electrocardiogram.

Characteristic	Number/value	Percentage / SD
Total study population	210	100
Age, mean ± SD (years)	57.49 ± 12.45	-
Age range (years)	30-90	-
18-40 years	25	11.9
40-60 years	105	50
>60 years	80	38.1
Male sex	141	67.1
Female sex	69	32.9
Common occupation: housewife	60	28.6
Common occupation: farmer	54	25.7
Common occupation: laborer	31	14.8
Hypertension	47	22.4
Diabetes mellitus	45	21.4
COPD	1	0.5
Smoking	48	22.9
Alcohol intake	10	4.8
Oral hypoglycemic agent use	45	21.4
ACE inhibitor/ARB use	10	4.7
Beta blocker use	6	2.8
Troponin-I positive	152	72.4
ST elevation on ECG	129	61.4
ST depression on ECG	36	17.1
T-wave inversion on ECG	137	65.2
Q waves on ECG	32	15.2
Hospital stay ≤5 days	159	75.7
Hospital stay >5 days	51	24.3
In-hospital mortality	10	4.8

Using an RDW-CV cut-off of 14.5%, 111 patients (52.9%) had elevated RDW-CV, while 99 patients (47.1%) had RDW-CV values ≤14.5% on Day 1. In comparison, RDW-SD was high in only 9 patients (4.3%) and low in 201 patients (95.7%). High RDW-CV was significantly associated with adverse in-hospital outcomes. Mortality occurred in nine patients in the high RDW-CV group and in one patient in the low RDW-CV group (p<0.05). RDW-SD did not show a statistically significant association with mortality (p=0.360), probably because very few patients had high RDW-SD (Table 2).

**Table 2:** RDW status and major clinical associations Data are presented as frequency and percentage. Mortality percentages were calculated using the respective RDW subgroup as the denominator. Elevated RDW-CV was defined as >14.5%, while values ≤14.5% were categorized as normal/low. Mortality refers to in-hospital mortality during the index hospitalization. Comparisons between groups were performed using the chi-square test or Fisher’s exact test where applicable. A p-value <0.05 was considered statistically significant. RDW-CV, red cell distribution width-coefficient of variation; RDW-SD, red cell distribution width-standard deviation; Troponin-I, cardiac troponin I.

Variable/group	Total patients, n (%)	Mortality, n/N (%)	Interpretation	p-value
RDW-CV low	99 (47.1)	1/99 (1.0)	Reference/low-risk group	<0.05
RDW-CV high	111 (52.9)	9/111 (8.1)	Higher mortality	<0.05
RDW-SD low	201 (95.7)	9/201 (4.5)	No significant association	0.36
RDW-SD high	9 (4.3)	1/9 (11.1)	No significant association	0.36
Troponin-I positive with low RDW-CV	64	-	Lower frequency than the high RDW-CV group	0.00179
Troponin-I positive with high RDW-CV	88	-	Significantly more frequent	0.00179

**Figure 2:**
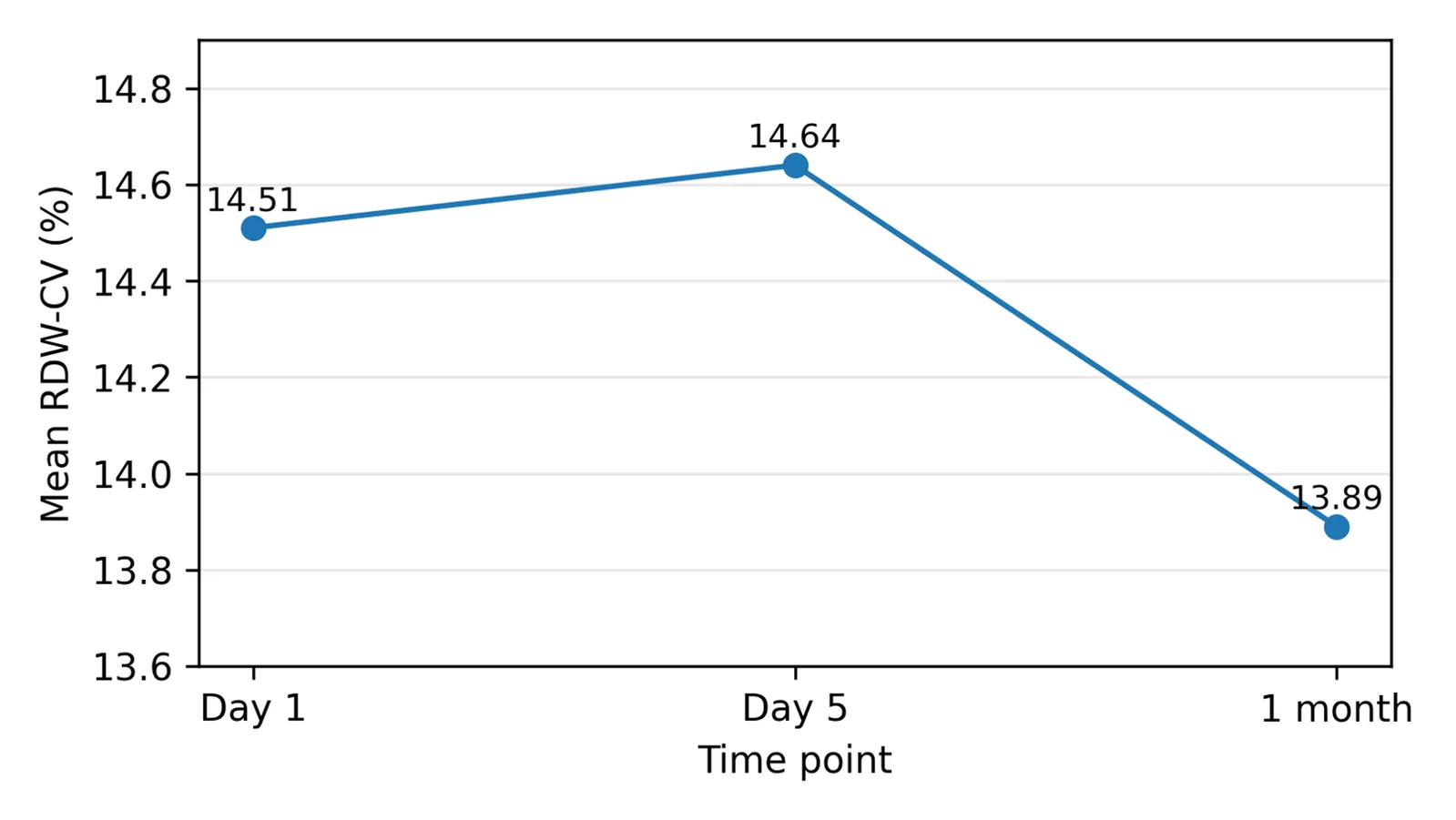
Trend of mean RDW-CV values over time RDW-CV remained elevated during the acute hospital phase, with a slight rise from Day 1 to Day 5, followed by a decline at the one-month follow-up. RDW-CV, red cell distribution width-coefficient of variation

Serial assessment demonstrated that RDW values remained elevated during the acute phase and declined during follow-up. Mean RDW-CV was 14.51 ± 2.22 on Day 1, increased slightly to 14.64 ± 2.25 on Day 5, and decreased to 13.89 ± 2.01 at one month. Mean RDW-SD showed a similar pattern, measuring 46.77 ± 4.80 on Day 1, 47.07 ± 5.03 on Day 5, and 45.70 ± 4.19 at one month. These serial changes were statistically significant. However, because RDW-CV is influenced by mean corpuscular volume (MCV) and the relationship between RDW-SD and mortality was not statistically significant, these changes should be interpreted as variations in RDW indices rather than definitive evidence of a reduction in true erythrocyte anisocytosis (Figure 2).

Gender-wise analysis showed that the mean RDW-CV on Day 1 was significantly higher in females (15.85 ± 1.67) than in males (13.85 ± 2.15; p<0.05). Mean RDW-SD on Day 1 was also higher among females (49.41 ± 4.53) compared with males (45.47 ± 4.38; p<0.05). No statistically significant difference in RDW-CV or RDW-SD was observed across different age groups. Patients with high RDW-CV had significantly higher pulse rate (89.08 ± 16.54/min) than those with low RDW-CV (83.83 ± 13.36/min; p<0.05), while hemoglobin, systolic blood pressure, diastolic blood pressure, WBC count, and serum creatinine did not differ significantly according to RDW-CV status.

ECG-based analysis demonstrated that RDW-CV was significantly associated with ST elevation (p=0.041) and T-wave inversion (p=0.0276), while no significant association was observed with ST depression (p=0.1452) or Q waves (p=0.2623). RDW-SD showed significant associations with ST depression (p=0.026), T-wave inversion (p=0.039), and Q waves (p=0.044), but not with ST elevation (p=0.303).

Clinical severity increased in parallel with RDW. Most patients belonged to Killip class I (149/210, 71.0%), followed by class II (26/210, 12.4%), class III (24/210, 11.4%), and class IV (11/210, 5.2%). Mean RDW-CV increased from 13.8 ± 2.1 in Killip class I to 16.6 ± 1.6 in class IV (p<0.001), and mean GRACE score increased from 97.13 ± 25.59 to 166.36 ± 21.08 across the same categories (p<0.0001). RDW-SD also demonstrated a significant increasing trend across Killip classes (p<0.001) (Table 3).

**Table 3:** RDW-CV and GRACE score according to Killip class Data are presented as mean ± SD unless otherwise specified. Comparisons of RDW-CV and GRACE scores across Killip classes were performed using one-way analysis of variance (ANOVA). A p-value <0.05 was considered statistically significant. RDW-CV, red cell distribution width-coefficient of variation; GRACE, Global Registry of Acute Coronary Events; SD, standard deviation.

Killip class	Patients, n (%)	RDW-CV, mean ± SD	GRACE score, mean ± SD
I	149 (71.0)	13.8 ± 2.1	97.13 ± 25.59
II	26 (12.4)	15.85 ± 1.6	107.23 ± 26.39
III	24 (11.4)	16.10 ± 1.5	136.00 ± 23.12
IV	11 (5.2)	16.6 ± 1.6	166.36 ± 21.08
p-value	-	<0.001	<0.0001

**Figure 3:**
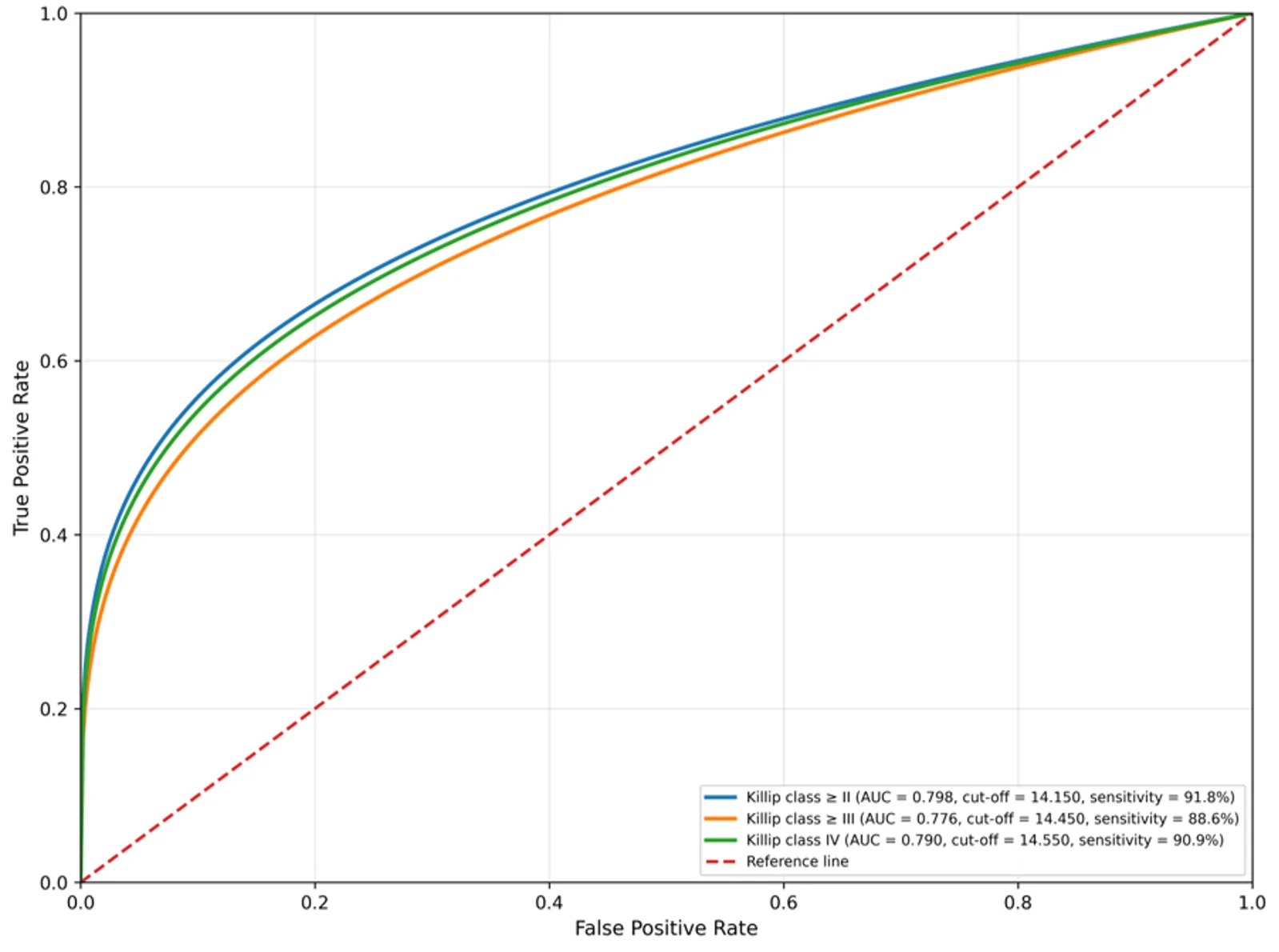
Receiver operating characteristic curve showing the predictive performance of RDW-CV for higher Killip class in patients with acute coronary syndrome RDW-CV showed acceptable discriminatory ability for predicting higher Killip class. The AUC was 0.798 for Killip class ≥ II, 0.776 for Killip class ≥ III, and 0.790 for Killip class IV. RDW-CV, red cell distribution width-coefficient of variation; AUC, area under the curve

Receiver operating characteristic (ROC) curve analysis further supported the prognostic utility of RDW for identifying patients with higher Killip class. For predicting Killip class II or above, RDW-CV showed an area under the curve (AUC) of 0.798 at a cut-off of 14.150, with a sensitivity of 91.8%. For Killip class III or above, the AUC was 0.776 at a cut-off of 14.450, with a sensitivity of 88.6%. For Killip class IV, the AUC was 0.790 at a cut-off of 14.550, with a sensitivity of 90.9%. RDW-SD also showed acceptable discriminatory ability, with AUC values of 0.868, 0.823, and 0.809 for Killip class II or above, class III or above, and class IV, respectively (Figure 3).

Spearman correlation analysis showed a significant positive correlation between GRACE score and RDW-CV on Day 1 (r=0.206, p=0.003), Day 5 (r=0.208, p=0.002), and at one month (r=0.186, p=0.007). GRACE score also correlated significantly with RDW-SD on Day 1 (r=0.247, p<0.0001), Day 5 (r=0.241, p<0.0001), and at one month (r=0.207, p=0.0025). Overall, elevated RDW, particularly RDW-CV, was associated with mortality, troponin-I positivity, ECG abnormalities, higher Killip class, higher GRACE score, and prolonged hospitalization, supporting its role as a simple prognostic marker in ACS.

## Discussion

ACS remains one of the most important causes of emergency medical admission and cardiovascular mortality worldwide. Early risk stratification is essential because ACS patients differ widely in clinical presentation, hemodynamic status, myocardial injury, and probability of adverse outcomes. In the present study, RDW was evaluated as a simple, inexpensive, and readily available hematological marker to assess prognosis in patients with ACS. The study included 210 patients, and the findings showed that RDW-CV was significantly associated with mortality and increased progressively with higher Killip class, suggesting its potential role as a prognostic marker in ACS.

In the present study, males were predominant. This finding is consistent with the established epidemiological pattern of ACS, where men are commonly affected more frequently than women, particularly in middle age. Prabhakaran et al. reported that cardiovascular disease is a major public health problem in India and that ischemic heart disease contributes substantially to cardiovascular mortality [4]. The higher male proportion in the present study may be related to greater exposure to conventional risk factors such as smoking, occupational stress, and lifestyle-related metabolic risk factors among males.

The mean age of the study population was 57.49 ± 12.45 years, and most patients belonged to the 40-60 years age group (105, 50.00%), followed by those above 60 years (80, 38.09%). This finding suggests that ACS was most common among middle-aged and elderly patients. Similar observations have been reported in Indian cardiovascular epidemiology studies, where ACS and coronary artery disease have been shown to occur at relatively younger ages compared with Western populations [12]. This younger age of presentation has important clinical implications, as ACS in the productive age group leads to increased socioeconomic burden, disability, and loss of quality-adjusted life years.

In the present study, hypertension was present in 47 patients (22.40%), diabetes mellitus in 45 patients (21.42%), and smoking in 48 patients (22.85%). These were the major baseline risk factors observed among ACS patients. These findings are consistent with previous reports identifying hypertension, diabetes mellitus, smoking, dyslipidemia, obesity, and sedentary lifestyle as major modifiable risk factors for coronary artery disease and ACS [13]. Hypertension contributes to endothelial dysfunction, vascular inflammation, and plaque instability, while diabetes mellitus accelerates atherosclerosis through insulin resistance, oxidative stress, and chronic low-grade inflammation. Smoking promotes thrombosis, endothelial injury, platelet activation, and coronary vasoconstriction, thereby increasing the risk of acute coronary events.

The present study found that RDW-CV was high in 111 patients (52.86%) and low in 99 patients (47.14%) on Day 1. RDW-SD was low in 201 patients (95.71%) and high in nine patients (4.29%). RDW is traditionally used to evaluate anemia, but recent evidence suggests it has prognostic value in cardiovascular diseases. Danese et al. reviewed the relationship between RDW and cardiovascular diseases. They reported that elevated RDW is associated with ACS, myocardial infarction, heart failure, stroke, peripheral artery disease, and adverse cardiovascular outcomes [7]. Bujak et al. also reported that RDW reflects multiple pathophysiological processes, including inflammation, oxidative stress, impaired erythropoiesis, and endothelial dysfunction, all of which are relevant to atherosclerosis and ACS [8].

A major finding of the present study was the significant association between high RDW-CV and mortality. Among patients with low RDW-CV, mortality occurred in one patient; among those with high RDW-CV, in nine patients. This association was statistically significant (p < 0.05), indicating that higher RDW-CV was associated with increased mortality in ACS patients. This finding is supported by Abrahan et al., who conducted a meta-analysis of 13 trials and 10,410 ACS patients, finding that low RDW was associated with significantly lower all-cause and cardiovascular mortality and a lower risk of major adverse cardiac events [14]. Similarly, Su et al. reported in a systematic review and meta-analysis that higher RDW levels were associated with increased mortality and cardiovascular events in patients with established coronary artery disease [9].

The present study showed that RDW-SD was not significantly associated with mortality (p=0.360), whereas RDW-CV demonstrated a significant association. RDW-CV is calculated relative to MCV and may therefore be influenced by changes in mean erythrocyte volume. In contrast, RDW-SD represents the absolute width of the erythrocyte volume-distribution curve and is less dependent on MCV. Consequently, the discordance between RDW-CV and RDW-SD indicates that the observed association of RDW-CV with mortality cannot be attributed conclusively to true anisocytosis alone. Furthermore, only nine patients had elevated RDW-SD, resulting in an imbalanced subgroup distribution and limited statistical power. Therefore, RDW-CV should be interpreted as a potentially useful prognostic hematological index rather than a direct and independent measure of anisocytosis.

The mean RDW-CV in the present study was 14.51 ± 2.22 on Day 1, increased slightly to 14.64 ± 2.25 on Day 5, and decreased to 13.89 ± 2.01 at the one-month follow-up. This trend suggests that RDW-CV remained elevated during the acute hospital phase and decreased during follow-up. The persistence of higher RDW during acute illness may reflect ongoing inflammatory activity, oxidative stress, and impaired erythrocyte turnover. The decrease at one month may indicate clinical stabilization following treatment and recovery from the acute inflammatory and ischemic phase. Turcato et al. reported that RDW is a valuable, simple, and inexpensive parameter for medium-term risk stratification after ACS [9]. Their findings support the clinical relevance of serial RDW assessment in patients with ACS.

Regarding ECG findings, T-wave inversion was present in 137 patients (65.23%) and absent in 73 patients (34.76%). RDW-CV showed a statistically significant association with T-wave inversion (p=0.0276). Among patients with high RDW-CV, 80 had T-wave inversion; among those with low RDW-CV, 57 had T-wave inversion. T-wave inversion is commonly associated with myocardial ischemia and may reflect ongoing ischemic burden. The significant association between high RDW-CV and T-wave inversion may suggest that increased RDW-CV is linked with greater ischemic stress in ACS patients. Tenekecioglu et al. reported that higher baseline RDW was associated with myocardial injury and elevated cardiac troponin I levels in patients with non-ST-elevation ACS [15].

The present study also found that RDW-CV increased progressively with a higher Killip class. Mean RDW-CV was 13.8 ± 2.1 in Killip Class 1, 15.85 ± 1.6 in Class 2, 16.10 ± 1.5 in Class 3, and 16.6 ± 1.6 in Class 4, with a statistically significant association (p < 0.001). Killip classification is a clinically useful tool for assessing heart failure severity in patients with myocardial infarction. A higher Killip class indicates more severe cardiac dysfunction and a worse prognosis. The progressive rise in RDW-CV with increasing Killip class in the present study suggests that RDW-CV may reflect hemodynamic severity and systemic stress in ACS. Uyarel et al. reported that elevated admission RDW was a prognostic marker in patients undergoing primary angioplasty for acute myocardial infarction [10].

The GRACE score also increased significantly with higher Killip class in the present study. The mean GRACE score was 97.13 ± 25.59 in Killip Class 1, 107.23 ± 26.39 in Class 2, 136.00 ± 23.12 in Class 3, and 166.36 ± 21.08 in Class 4, with p < 0.0001. This confirms that increasing clinical severity was associated with a higher predicted risk of adverse outcomes. Granger et al. developed the GRACE risk model and demonstrated its utility for predicting in-hospital mortality among ACS patients [10]. In the present study, RDW-CV also showed a positive correlation with GRACE score at Day 1, Day 5, and 1 month, indicating that RDW-CV may complement established risk stratification tools.

ROC analysis in the present study showed that RDW-CV had good predictive ability for higher Killip classes. The AUC was 0.798 for predicting Killip Class ≥2, 0.776 for Killip Class ≥3, and 0.790 for Killip Class 4. These findings suggest that RDW-CV has acceptable discriminatory ability for identifying patients with a more severe clinical presentation. Similar evidence has been reported in previous cardiovascular literature, where RDW has been shown to predict mortality, major adverse cardiac events, and poor outcomes in coronary artery disease and ACS populations [5,6,9]. Because RDW is part of routine complete blood count testing, it may be especially useful in settings where advanced biomarkers or invasive assessments are not immediately available. These ROC-derived cut-offs are exploratory and specific to the present study population and laboratory method; they should not be interpreted as universally applicable clinical thresholds without external validation.

The biological plausibility for the association between RDW and poor ACS outcome is strong. Inflammation suppresses erythropoietin activity, alters iron metabolism, and releases immature erythrocytes into circulation, leading to anisocytosis and increased RDW [3,4]. Oxidative stress reduces red blood cell deformability and survival, while endothelial dysfunction contributes to plaque instability and thrombosis. These mechanisms are central to the pathophysiology of ACS. Therefore, elevated RDW-CV may reflect a combination of systemic inflammation, oxidative stress, altered erythropoiesis, changes in erythrocyte volume, and other underlying clinical factors. However, the present findings do not establish whether true anisocytosis itself contributes directly to adverse outcomes in ACS.

Limitations

The present study has certain limitations. Its single-center setting, limited number of mortality events, and short follow-up may restrict the precision and generalizability of the findings. Long-term mortality and major adverse cardiovascular events were not evaluated, and multivariable analysis could not be performed. Although major conditions known to affect RDW were excluded, unmeasured factors, including nutritional deficiencies, subclinical inflammation, dyslipidemia, anemia, and lifestyle, may have caused residual confounding. Iron profile, vitamin B12, folate, CRP, and complete lipid-profile parameters were not systematically assessed. Moreover, RDW-CV is influenced by MCV, whereas RDW-SD more directly reflects erythrocyte-size variation. The discordant mortality findings for these indices, absence of MCV-stratified analysis and peripheral-smear confirmation, and small number of patients with elevated RDW-SD prevent attributing the observed association specifically to anisocytosis. Larger multicentric studies incorporating RDW-CV, RDW-SD, MCV, relevant confounders, and adjusted analyses are required.

## Conclusions

The present study demonstrated that elevated RDW-CV was associated with mortality and greater clinical severity in patients with ACS. However, RDW-SD was not significantly associated with mortality, and the findings do not establish that true erythrocyte anisocytosis independently influences ACS outcomes. RDW-CV may therefore be considered an inexpensive and readily available adjunctive prognostic index, but it should be interpreted alongside MCV, RDW-SD, clinical findings, ECG changes, cardiac biomarkers, Killip class, and GRACE score. Further appropriately powered studies are required before definitive clinical conclusions can be drawn.

## Figures and Tables

**Figure 4:**
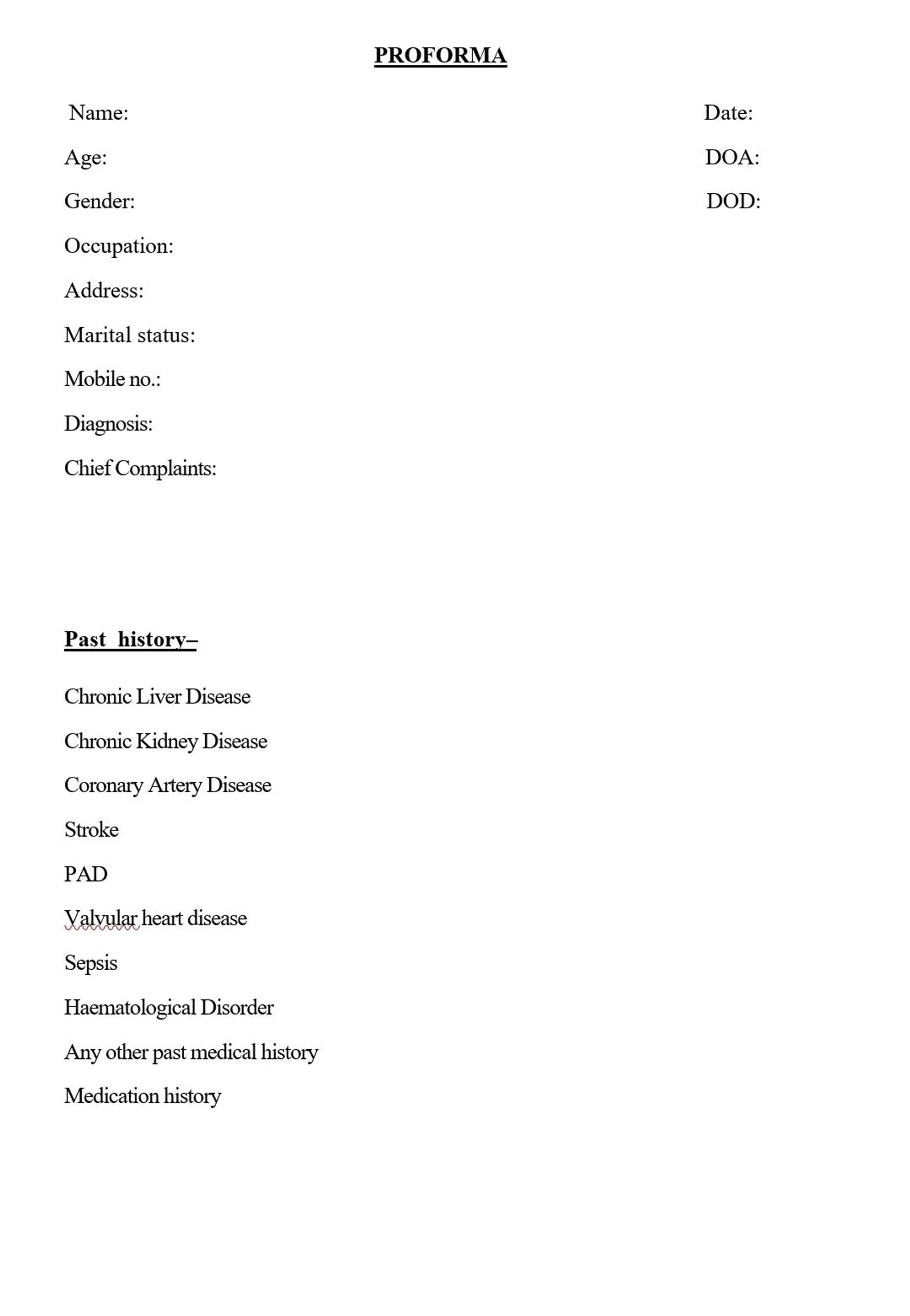
Proforma Page 1

**Figure 5:**
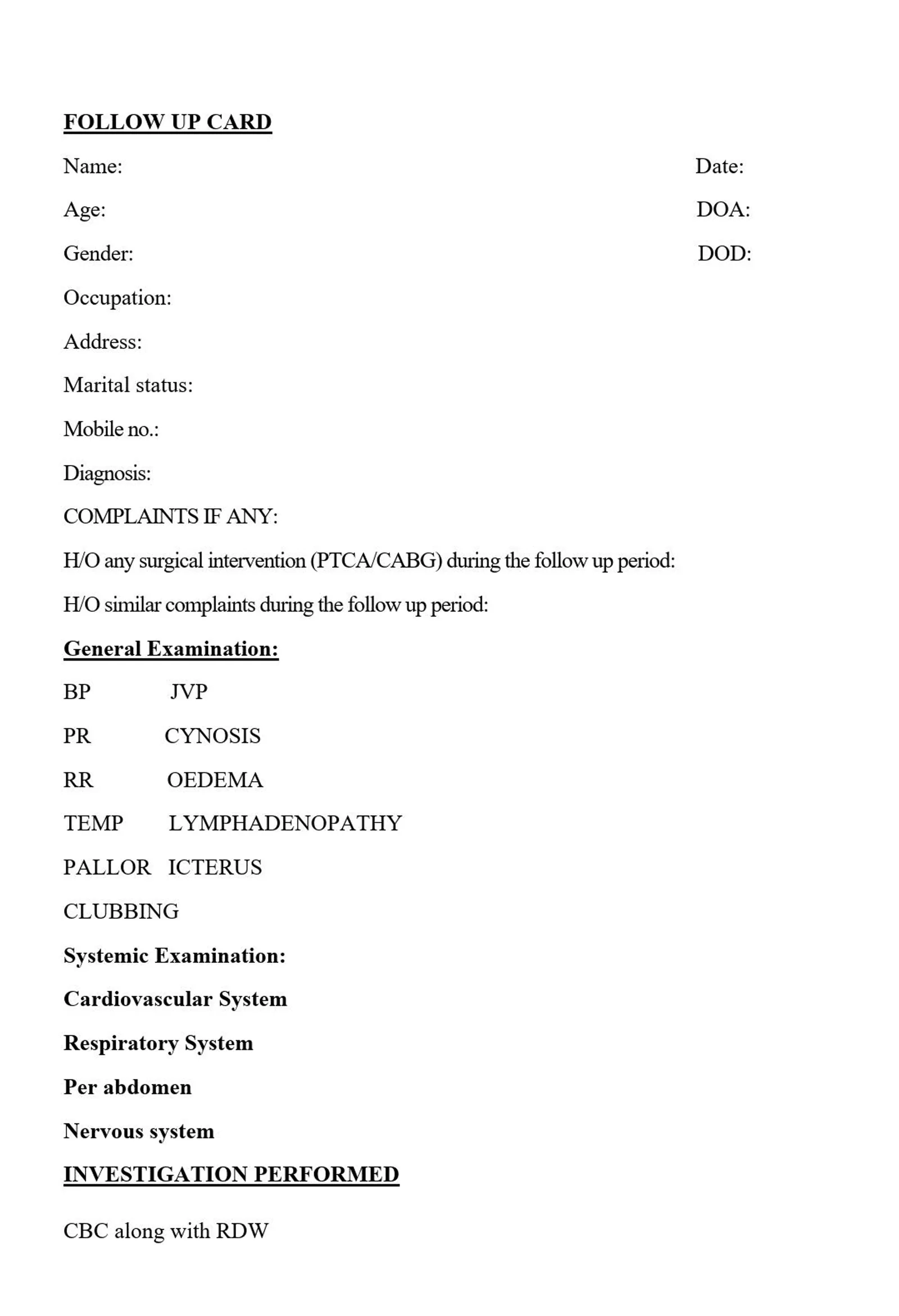
Proforma Page 2

**Figure 6:**
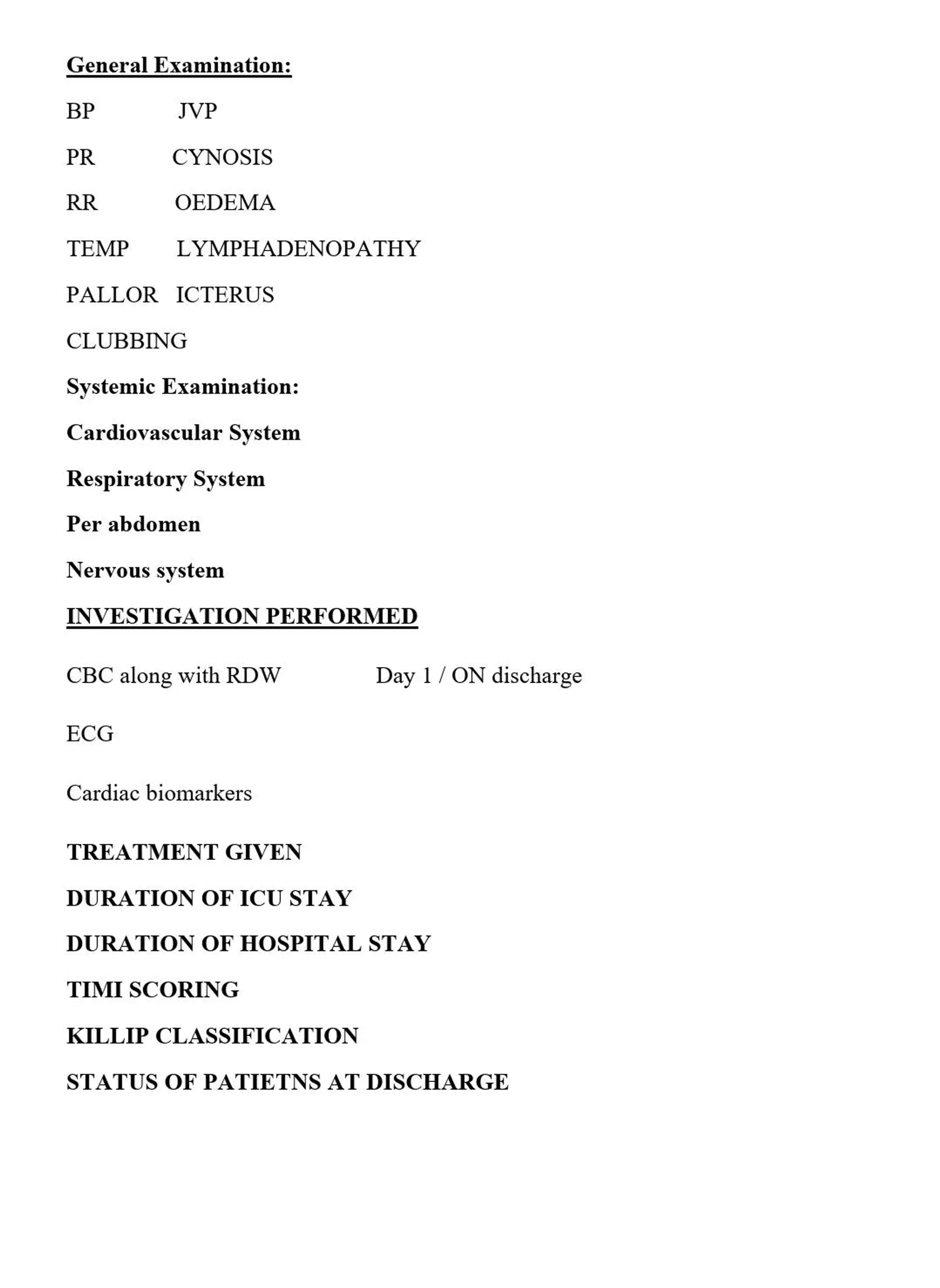
Proforma Page 3

## Appendices

Appendix A

Restructured Proforma and Case Record Format for Recording Data
